# Effects on mental health of a UK welfare reform, Universal Credit: a longitudinal controlled study

**DOI:** 10.1016/S2468-2667(20)30026-8

**Published:** 2020-02-27

**Authors:** Sophie Wickham, Lee Bentley, Tanith Rose, Margaret Whitehead, David Taylor-Robinson, Ben Barr

**Affiliations:** a Department of Public Health and Policy, University of Liverpool, Liverpool, UK; b Section of Epidemiology, Department of Public Health, University of Copenhagen, Copenhagen, Denmark

## Abstract

**Background:**

Universal Credit, a welfare benefit reform in the UK, began to replace six existing benefit schemes in April, 2013, starting with the income-based Job Seekers Allowance. We aimed to determine the effects on mental health of the introduction of Universal Credit.

**Methods:**

In this longitudinal controlled study, we linked 197 111 observations from 52 187 individuals of working age (16–64 years) in England, Wales, and Scotland who participated in the Understanding Society UK Longitudinal Household Panel Study between 2009 and 2018 with administrative data on the month when Universal Credit was introduced into the area in which each respondent lived. We included participants who had data on employment status, local authority area of residence, psychological distress, and confounding variables. We excluded individuals from Northern Ireland and people out of work with a disability. We used difference-in-differences analysis of this nationally representative, longitudinal, household survey and separated respondents into two groups: unemployed people who were eligible for Universal Credit (intervention group) and people who were not unemployed and therefore would not have generally been eligible for Universal Credit (comparison group). Using the phased roll-out of Universal Credit, we compared the change in psychological distress (self-reported via General Health Questionnaire-12) between the intervention group and the comparison group over time as the reform was introduced in the area in which each respondent lived. We defined clinically significant psychological distress as a score of greater than 3 on the General Health Questionnaire-12. We tested whether there were differential effects across subgroups (age, sex, and education).

**Findings:**

The prevalence of psychological distress increased in the intervention group by 6·57 percentage points (95% CI 1·69–11·42) after the introduction of Universal Credit relative to the comparison group, after accounting for potential confounders. We estimate that between April 29, 2013, and Dec 31, 2018, an additional 63 674 (95% CI 10 042–117 307) unemployed people will have experienced levels of psychological distress that are clinically significant due to the introduction of Universal Credit; 21 760 of these individuals might reach the diagnostic threshold for depression.

**Interpretation:**

Our findings suggest that the introduction of Universal Credit led to an increase in psychological distress, a measure of mental health difficulties, among those affected by the policy. Future changes to government welfare systems should be evaluated not only on a fiscal basis but on their potential to affect health and wellbeing.

**Funding:**

Wellcome Trust, UK National Institute for Health Research, and Medical Research Council.

## Introduction

Mental health in the UK has deteriorated in the past two decades and there is evidence that welfare reforms have contributed to this decline.^1^, ^2^ In 2013, the UK introduced a major change to its welfare system and began replacing several existing benefit schemes with a new benefit called Universal Credit. Doctors have raised concerns that this reform is harming health and increasing the workload of general practitioners.^3^

**Figure 1:**
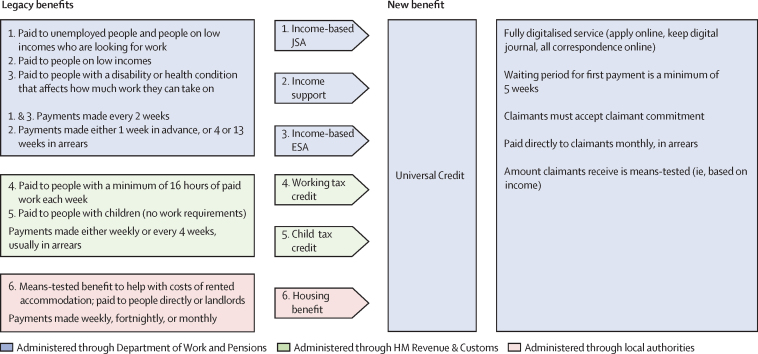
Outline of Universal Credit, and the legacy welfare benefits it replaces in the UK JSA=Job Seekers Allowance. ESA=Employment and Support Allowance.

Universal Credit replaces six welfare benefits covering housing and living costs for people facing adversity, such as unemployment, disabilities, and low-paid employment (figure 1). It was introduced at different times in different parts of the UK, starting in the northwest of England in April, 2013, and was implemented in stages, initially affecting unemployed individuals and then people in work who were receiving tax credits. By the end of 2018, all parts of the UK had introduced Universal Credit for unemployed people and 1·6 million people were receiving Universal Credit, including 72·9% of all unemployed people in the UK and 1·8% of all employed people (see video for maps of roll-out).^4^, ^5^ Universal Credit introduced various features that differed from previous benefit schemes, including a fully digitised service, paying benefits directly to claimants, paying monthly in arrears rather than prospectively each week, increased conditionality, and reduced amounts paid to some claimant groups.^6^ Universal Credit was intended to provide greater incentives for claimants to enter employment and to ensure that the receipt of benefits “maximises claimants' responsibility and self-sufficiency”^6^ and that it mimics work and receipt of a salary (ie, by paying claimants monthly in arrears and paying all monies direct to the claimant).^7^

**Research in context**

**Evidence before this study**

We searched MEDLINE, PubMed, Scopus, PsycINFO, and Social Science Citation Index for articles and grey literature published in English between April 1, 2013, and Aug 15, 2019, using the search terms “universal credit” AND “mental health” OR “wellbeing” OR “well-being” OR “depress*” OR “anxiety” OR “psychiatric disorder*” OR “common mental disorder*” OR “psych* morbidity”. Our search yielded three commentary pieces and two qualitative research articles. The commentary pieces were authored by doctors who reported that changes to the welfare benefit system had increased their workload, and observed more patients attending general practitioners with mental health problems triggered or made worse by Universal Credit. The two qualitative research articles report that the Universal Credit claims process, conditionality, and sanction threats exacerbated long-term health conditions and negatively affected participants' mental health. Stringent conditionality of Universal Credit was identified by one article as the main source of negative wellbeing, while failing to improve employment outcomes. No empirical studies included in our search have evaluated the effects on mental health of the introduction of Universal Credit.

**Added value of this study**

We used a quasi-experimental study design, which took advantage of the phased roll-out of Universal Credit across areas in the UK, to investigate whether the introduction of Universal Credit in an area was associated with an increase in mental health problems among unemployed people eligible for Universal Credit, compared with a comparison group who were not eligible. Our findings suggest that the introduction of Universal Credit, a major UK welfare reform, has led to an increase of 6·57 percentage points (95% CI 1·69–11·42) in psychological distress among unemployed individuals affected by the policy. The number of unemployed people moving on to Universal Credit is large, with our estimates suggesting that 63 674 people have been negatively affected by this welfare policy change, of whom 21 760 might reach levels that are diagnostically depression, indicating the potential clinical significance of our findings. We found no evidence that Universal Credit exposure was associated with moving into employment.

**Implications of all the available evidence**

It is crucial that, when assessing the costs and benefits of new welfare policies, policy makers take into account the potential health consequences. To date, the national evaluation framework for Universal Credit only includes an assessment of labour market outcomes. There is no plan to assess the effects on health and wellbeing. Given the evidence for the adverse effects on health of welfare change—as suggested by this study and previous analyses—it is crucial that a robust health impacts assessment exists within any evaluation for welfare change, including Universal Credit, and that health effects are considered when redesigning welfare systems.

Universal Credit has been the subject of a great deal of controversy in the UK, with reports of long delays in payments and increased use of sanctions whereby claimants lose part or all of their benefits for not meeting conditions, such as looking for work. There have been anecdotal reports that the policy has increased food bank usage, mental health difficulties amongst claimants, and consultations in general practices.^3^, ^7^, ^8^, ^9^, ^10^, ^11^ Several qualitative studies^11^, ^12^ have concluded that Universal Credit adversely affected claimants' financial security, driving people further into poverty and food insecurity, worsening physical and mental health, and negatively affecting their social and family lives and employment prospects. They found that managing the Universal Credit claims process and increased conditionality, combined with the threat of sanctions, exacerbated long-term health conditions and negatively affected participants' mental health such that some had considered suicide.^12^

Understanding the mental health impact of this major welfare reform is of international importance for health professionals who are responding to the rising mental health needs of populations and for policy makers who are deciding the most appropriate and effective approaches to welfare policy changes. To our knowledge no empirical studies have evaluated the effects on mental health of the introduction of Universal Credit. We aimed to use a quasi-experimental study design, which took advantage of the phased roll-out of Universal Credit across areas in the UK, to investigate whether the introduction of Universal Credit in an area was associated with an increase in mental health problems among unemployed people eligible for Universal Credit, compared with a comparison group who were not eligible.

## Methods

### Study design

We used data from the Understanding Society UK Longitudinal Household Panel Study (USLHPS).^13^ USLHPS is a nationally representative, longitudinal panel survey based on a stratified clustered random sample of 40 000 households from the four UK countries done between 2009 and 2018 and includes eight waves of data, where each individual might have participated up to eight times. Each wave of data was collected over a period of 3 years (eg, 2016–18), with most data being collected over 1 year (eg, 2017) and small proportions being collected either side of this. For weighting purposes, we used wave years as opposed to true years as using true years would bias the findings, because the weighting variable in Understanding Society is specified by wave not true year. Data were collected by trained interviewers using face-to-face surveys. A special licence version of the data was used, which included identifiers of the local authority in which respondents lived. We used these data to match interview responses to a dataset indicating whether Universal Credit was available in their area at the date of interview, using data obtained from the UK Department of Work and Pensions.^4^, ^5^

### Participants

We included participants of working age (16–64 years) who had data on employment status, local authority area of residence, our outcome measure (psychological distress), and confounding variables. We excluded individuals from Northern Ireland because data on the availability of Universal Credit were not available for these local authorities. We also excluded people out of work with a disability (appendix p 1) because they would not have been affected by the introduction of Universal Credit, but would have been affected by changes to disability benefits—which have been shown to have adverse mental health effects during this time.^1^ A flowchart of participants and details of the study sample can be found in the appendix (p 2).

### Procedures

Individuals were assigned to the intervention group in a given wave if they identified as unemployed. We restricted the criteria to only unemployed individuals because they were the first claimant group to be eligible for Universal Credit, with all new claims from unemployed individuals generally being eligible for Universal Credit after it was introduced in an area (appendix p 3). Unemployment was self-reported in the USLHPS. Individuals were asked “Which of these best describes your current employment situation?” and given 12 possible options: employed, self-employed, unemployed, retired, on maternity leave, looking after family, full-time student, long-term sick or disabled, on a government training scheme, unpaid worker in family, working in an apprenticeship, or doing something else. Individuals were assigned to the comparison group in a given wave if they identified as anything other than unemployed. A small proportion (<2%) of the comparison group would have become eligible for Universal Credit during the roll-out process (eg, employed people on tax credits in some areas); however, it was not possible to identify these respondents in the data. Their inclusion in the comparison group provides a more conservative estimate of the intervention effect. If survey respondents entered unemployment in subsequent waves, they were assigned to the intervention group for that wave; if respondents left unemployment in subsequent waves, they were assigned to the comparison group for that wave.

Given that Universal Credit was implemented through Job Centre Plus areas, which map onto local authorities for each respondent, we created a dummy variable that was 1 if Universal Credit had been introduced in their local authority area and 0 otherwise. An area was defined as having introduced Universal Credit if there were one or more claimants receiving Universal Credit in that local authority area. By the end of the study period, all areas had introduced Universal Credit for unemployed people (see video for map of roll-out).

### Outcomes

Our primary outcome was self-reported psychological distress using the General Health Questionnaire-12 (GHQ-12). The GHQ-12 is a unidimensional measure of general psychological distress,^14^ including experiences of depression.^15^, ^16^ We used a dichotomised score, where 0–3 indicated no difficulties (scored as 0) and 4–12 indicated psychological distress (scored as 1).^17^ As a secondary outcome, we included the GHQ-12 score as a continuous measure.

To examine whether our findings were sensitive to the mental health measure used, we also analysed the effect of the intervention on the mental component summary of the 12-item Short Form Health Survey (SF-12). This validated screening tool measures recent and active depression in the general population, with scores from 0 to 100 wherein higher scores indicate better mental health.^18^

### Statistical analysis

We used difference-in-differences analysis to estimate the effects on mental health of the introduction of Universal Credit. This longitudinal method allowed us to compare the change in outcomes in the intervention population with the change in outcomes in the comparison population, before and after Universal Credit was introduced **a**cross the UK. The difference-in-differences method controls for all time-invariant differences between the intervention and comparison populations. The key assumption is that trends in the outcome in these two groups would have been parallel in the absence of the intervention. If this assumption is true, the difference between the change in the outcomes between the two populations provides an unbiased estimate of the interventions effect (known as the parallel trends assumption). We examined whether the trends were parallel before the intervention—graphically and using regression models—to compare trends in the outcomes of interest between the intervention and comparison groups in the pre-intervention period.

For our primary outcome, we estimated the difference-in-differences parameter by fitting a multivariable logistic regression model using a complete case sample, including an interaction term between the policy exposure period and the intervention group (appendix pp 4–5) to the longitudinal individual-level data. Although the intervention and comparison groups are not comparable in terms of mental health experiences before Universal Credit, this in itself does not introduce bias in the difference-in-differences analysis. We used robust clustered standard errors to account for the area-level clustering and the longitudinal nature of the data, and included survey weights to account for non-response. To account for potential household-level clustering within individuals, we included household identification numbers as the primary sampling unit. To account for potential demographic and socioeconomic changes that could confound the result, we included participants' country of residence, age, sex, education status, and marital status. We used this model to estimate the absolute change in the prevalence of psychological distress in the intervention group versus the comparison group (the difference-in-differences parameter) using the margins command in Stata (version 14; College Station, TX, USA). To investigate whether there were differential effects across subgroups, we tested the interaction of age with the intervention by period interaction term. We repeated this analysis for sex and education in three separate logistic regression models.

We repeated the primary outcome (GHQ-12) difference-in differences analysis for two other outcomes: GHQ-12 score as a continuous measure and mental component summary of the SF-12. For models with a continuous outcome, we used a linear rather than logistic regression model. Although the residuals in these models are likely to diverge from normal distribution, we used robust standard errors that are consistent even when data departs from normality. Additionally, linear models provide a robust estimate of the difference-in-differences estimator even when the data are not normally distributed.^19^

In an additional analysis, we also investigated whether the introduction of Universal Credit led to an increase in employment for those that were unemployed after the introduction of Universal Credit, because this outcome was a stated aim of the policy. We also did a post-hoc analysis to assess the effect of Universal Credit on physical health using the SF-12 physical component summary.

We did various robustness tests to investigate whether our results were sensitive to model specification.^20^ We tested whether the trends in all our outcomes were parallel before the intervention. Given that the model with a logit link function could bias the results because it assumes non-linear trends, we repeated our main analysis using a linear probability rather than logistic model. We replicated our analysis taking out those who identified as employed or self-employed from the comparison sample, because trends in the psychological distress of the employed might have diverged from people out of work, which could bias the results. To test if our analysis could be biased by duration in unemployment or baseline psychological distress, we restricted the main analysis to only include people who were newly unemployed and who had new onset of psychological distress to see if becoming newly unemployed has a greater adverse effect on new experiences of psychological distress—than psychological distress accumulated during time in unemployment—after the introduction of Universal Credit. To determine whether differential attrition rates between intervention and comparison groups had biased the results (creating a balanced panel), we repeated the main analysis to only include people who participated in all eight waves of the survey. Finally, we repeated the main analysis using multiple imputations to account for potential bias in the missing data.

### Role of the funding source

The funders of the study had no role in study design, data collection, data analysis, data interpretation, or the writing of the report. The corresponding author had full access to all of the data in the study and had final responsibility for the decision to submit for publication.

## Results

We included 197 111 observations from 52 187 individuals of working age (16–64 years) in England, Wales, and Scotland who participated in the USLHPS between 2009 and 2018. We linked these data with administrative data on the month when Universal Credit was introduced into the area in which each respondent lived. Table 1 shows baseline characteristics of the intervention and comparison groups in the year before Universal Credit was introduced. Psychological distress was more prevalent in the intervention group than in the comparison group before the policy change. Participants in the intervention group were more likely to be male, unmarried, and younger (table 1), with lower educational qualifications than the comparison group. The difference-in-differences method accounts for these fixed differences in the analysis.

**Table 1:** Baseline characteristics in the year before Universal Credit was introduced, unweighted (n=22 896)

	**Intervention group**	**Comparison group**	**p value**
**General Health Questionnaire-12**			
Psychological distress	453 (35·8%)	3800 (17·6%)	..
No psychological distress	814 (64·2%)	17 829 (82·4%)	<0·0001
**Country of residence**			
England	1097 (86·6%)	18 679 (86·4%)	..
Wales	81 (6·4%)	1272 (5·9%)	..
Scotland	89 (7·0%)	1678 (7·8%)	0·503
**Sex**			
Male	620 (48·9%)	9456 (43·7%)	..
Female	647 (51·1%)	12 173 (56·3%)	<0·0001
**Age, years**			
16–24	367 (29·0%)	3903 (18·0%)	..
25–34	272 (21·5%)	3870 (17·9%)	..
35–44	223 (17·6%)	4680 (21·6%)	..
45–54	240 (18·9%)	5069 (23·4%)	..
55–64	165 (13·0%)	4107 (19·0%)	<0·0001
**Education level attained**			
Degree or higher	340 (26·8%)	8943 (41·3%)	..
GCSE, A levels, or equivalent	583 (46·0%)	9327 (43·1%)	..
Below GCSE or other	344 (27·2%)	3359 (15·5%)	<0·0001
**Marital status**			
Unmarried	918 (72·5%)	10 752 (49·7%)	..
Married or in a civil partnership	349 (27·5%)	10 877 (50·3%)	<0·0001

Data are n (%). We defined clinically significant psychological distress as a score of greater than 3 on the General Health Questionnaire-12.

**Figure 2:**
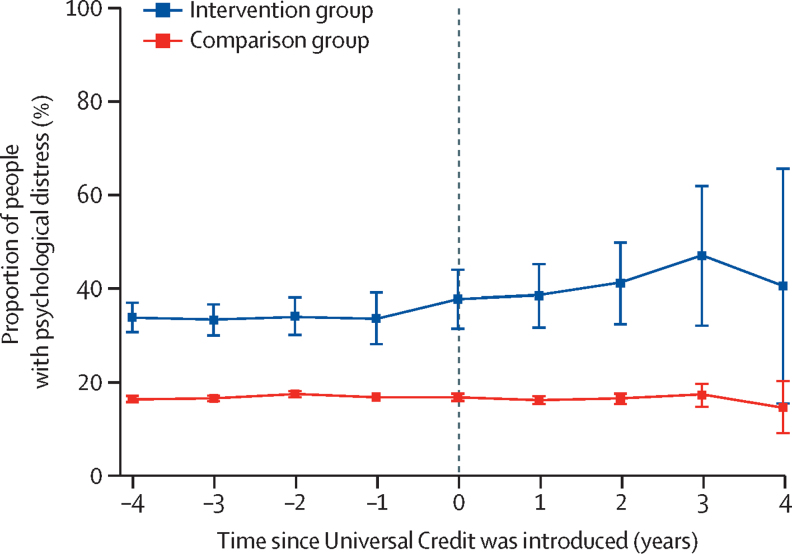
Psychological distress in the intervention and comparison groups before and after universal credit was introduced Error bars show 95% CI.

Figure 2 shows the trend in the proportion of people with psychological distress in the intervention and comparison groups before and after Universal Credit was introduced. Although the intervention group (unemployed individuals) had a higher prevalence than the comparison group before the intervention, this gap remained constant over time and the trends are parallel up to the point when Universal Credit is introduced. Regression analysis results are given in the appendix (p 6). When the policy change was introduced, the prevalence of psychological distress started to increase among those eligible for Universal Credit; however, the prevalence remained constant for people not affected by the change (comparison group).

Table 2 shows the difference-in-differences estimates from the multivariable regression models for our three mental health outcomes. For all three outcomes, mental health deteriorated after the introduction of Universal Credit among the intervention group, relative to those in the comparison group. The prevalence of psychological distress in the intervention group relative to the comparison group increased by 6·57 percentage points (95% CI 1·69 to 11·42); the average score on the GHQ-12 scale increased by 1·28 points (0·61 to 1·95), and the average score on the SF-12 mental component summary decreased by 1·45 points (−2·58 to −0·32). In relative terms, the increase of 6·57 percentage points in the prevalence of psychological distress is equivalent to a 21% increase in psychological distress relative to the weighted baseline prevalence in the intervention group of 32% (ie, 6·57 × 100/32=21). Similarly, relative to baseline levels, the absolute increase in GHQ-12 score is equivalent to a 10% reduction in average GHQ-12 score (based on weighted baseline prevalence in the intervention group of 13%) and, relative to baseline scores, a 3% reduction in average SF-12 mental component summary score (based on weighted baseline prevalence in the intervention group of 46%). Full results are given in the appendix (p 7).

**Table 2:** Difference-in-differences estimates

	**Estimate**	**95% CI**	**p value**
Odds ratio, change in odds of psychological distress associated with the intervention (GHQ-12 caseness)	1·38	1·11 to 1·72	0·003
Percentage point change in prevalence of psychological distress (GHQ-12 caseness)	6·57	1·69 to 11·42	0·008
Change in continuous GHQ-12 score	1·28	0·61 to 1·95	<0·0001
Change in mental health component of SF-12 score	−1·45	−2·58 to −0·32	0·01

Data are estimates of the change in outcomes among people eligible for Universal Credit after its introduction relative to the change in outcomes among those not affected by the policy change. Model based on equations is shown in the appendix (pp 4–5), with adjustment for country, age, sex, education level, and marital status. Full model results are given in the appendix (pp 7–8). GHQ-12=General Health Questionnaire-12. SF-12=12-item Short Form Health Survey. GHQ-12 Caseness=GHQ-12 score of greater than 3.

Based on the increase of 6·57 percentage points in the prevalence of psychological distress among the intervention group (ie, unemployed individuals), we estimate that between 2013 and 2018 the introduction of Universal Credit might have led to an additional 63 674 (95% CI 10 042–117 307) unemployed people experiencing psychological distress. Of these individuals, we estimate 21 760 might reach the diagnostic threshold for depression.

In post-hoc analysis, we tested if there was an effect on physical health, using the change in SF-12 physical component score. The introduction of Universal Credit did not affect the physical health of unemployed people (difference-in-differences estimator −0·6 [95% CI −1·5 to 0·3], p=0·17). We also tested if there was an increase in the number of participants transitioning from unemployment into work in the intervention group after the introduction of Universal Credit relative to the comparison group; the reform had no effect on employment (odds ratio 1·0 [95% CI 0·7–1·4], p=0·996).

Testing the robustness of our analysis—ie, by repeating it using a linear rather than a logistic regression model, removing employed people from the comparison sample, restricting analyses to those with only new onset of unemployment and psychological distress, using a balanced panel, and using multiple imputations—produced similar results (appendix pp 9–11). There was no evidence of any interaction between the difference-in-differences estimator and age group, sex, or educational group (ie, the policy did not have a differential effect across these subgroups).

## Discussion

Our longitudinal analysis suggests that the introduction of Universal Credit, a major UK welfare reform, has led to an increase in psychological distress of 6·57 percentage points among unemployed individuals exposed to the policy. The number of unemployed people moving on to Universal Credit is large, with our estimates suggesting that 63 674 unemployed people have been negatively affected by this welfare policy change. We found no evidence that Universal Credit exposure was associated with moving into employment.

To contextualise our findings, a study^21^ investigating the diagnostic ability of the GHQ-12 for mental health diagnosis in primary care found a positive predictive value of 34% based on the cutoff scores we have used in our main analysis. We estimate that, of the 63 674 unemployed individuals that might experience psychological distress as a result of the introduction of Universal Credit, 21 760 of these might reach the diagnostic threshold for depression—indicating the potential clinical significance of our findings. Therefore, we suggest that although the effect sizes in our study are moderate, the potential for psychological impact are substantial owing to the nature of policy implementation, which is on a national scale.

Previous research has suggested that austerity, welfare reforms, and greater conditionality have had adverse effects on the health and benefit mental health of claimants.^1^, ^2^, ^8^, ^9^, ^12^, ^22^, ^23^, ^24^, ^25^ Qualitative evidence has found increased conditionality, threat of sanctions, and suicidal thoughts associated with Universal Credit.^12^ Our research contributes to this existing body of knowledge and the sparse longitudinal evidence showing the significant mental health effects of moving onto Universal Credit for unemployed individuals.

Although mental health—as measured by the GHQ-12 and the SF-12 mental component summary—was our primary outcome, we also did a post-hoc analysis to assess the effect of Universal Credit on physical health using the SF-12 physical component summary. We found no effect on physical health.

This study has several strengths. First, we used a natural policy experiment approach, using difference-in-differences methodology to take advantage of the variation in policy exposure across different areas at different times, which can control for all time-invariant differences between the intervention and comparison populations. Second, our study has a large sample size providing a reasonable power to detect the effect of the intervention. Third, we show similar effects when using different measures of mental health (ie, GHQ *vs* SF-12 mental component summary). Finally, the conclusions of our analysis are strengthened by the robustness tests showing similar effects from different model specifications.

This study has some limitations. First, we use reported unemployment to define eligibility. Although unemployed individuals were the first group to become eligible for Universal Credit, not all unemployed people immediately moved onto Universal Credit when it was first introduced into an area. Initially, eligible people were only moved onto Universal Credit when they were making a new claim or had a change in circumstances. This practice meant that only 72·9% of unemployed individuals (∼990 000 people) were receiving Universal Credit after its introduction and 27·1% (∼370 000) remained on legacy benefits. Similarly, because Universal Credit was gradually rolled out to claimants of other benefit types, some participants in the comparison group will have become eligible over the course of our analysis; however, we estimate that only a small proportion (<2%) of the comparison group would have been affected. Our analysis is therefore likely to underestimate the effect of the policy, reflecting our conservative approach.

Second, we used self-reported measures of unemployment and mental health. It is possible that the introduction of Universal Credit might have influenced how people classify themselves with regards to employment status, which could have biased our findings. It is possible that people under-reported their experiences of unemployment, which would mean our findings underestimate the true policy effect. Although the mental health measures we used have been validated against clinical assessment, uncertainty remains as to the extent to which these measures define clinically relevant populations.^16^, ^17^, ^18^ We therefore used a combination of measures, utilising both validated cutoffs and continuous measures.

Third, missing data and attrition are ubiquitous problems in longitudinal datasets and natural policy methodologies. We used a complete case analysis based on unbalanced panel data, which might have biased the main findings if missingness was not random. However, repeating the analysis to only include people who participated in all eight waves of the survey to account for differential attrition rates between intervention and comparison groups resulted in similar results, finding a higher estimate of impact (10 percentage point increase in psychological distress). We also repeated the analysis using multiple imputations to account for bias in missing data, and again found similar results.

Finally, we were only able to capture the effect of Universal Credit on psychological distress in unemployed individuals, as these people were the most likely to be affected over the study period. Further investigation is warranted in other groups of people who might be affected by the implementation of Universal Credit—for example, employed people who received legacy working tax credits, and people who received legacy child tax credits—because it is important that the impact on all groups of society be researched and evaluated. Moreover, it is important to acknowledge that Universal Credit has been implemented within broader welfare changes (appendix p 3) that might have contributed to the psychological distress experienced by our research sample; for example, the under-occupancy penalty (bedroom tax), abolition of discretionary social funds, introduction of the benefit cap, and the introduction of the benefit freeze. Our results have several implications for policy. We suggest that the introduction of Universal Credit had a substantial negative effect on the mental health of unemployed individuals, adding to the growing body of evidence showing that restricting access to and reducing the adequacy of welfare benefits has a negative impact on health.^1^, ^25^ Given that 64% of households in the UK receive some kind of welfare benefit,^26^ changes to the welfare system—even those that have small individual effects—can have major implications for population health. Moreover, our findings of no differential effect of Universal Credit on the basis of education, a measure of socioeconomic status, is particularly interesting. Often, the adverse effect of welfare policy changes can be greater for more disadvantaged individuals, but we detected harmful effects of similar magnitude across all social groups. However, although the effect is similar for differential educational groups, because individuals with a lower attainment of education are more likely to be exposed to the policy (because they are more likely to become unemployed), the mental health of this group will be more affected by the policy change overall, potentially widening health inequalities. It is possible that our analysis might not have had sufficient power to detect such an effect. Primary care physicians are often one of the first groups to recognise these consequences,^3^ and they can play an important role in advocating for welfare policies that better promote health. Our evidence provides support for the growing calls for Universal Credit to be fundamentally modified to reduce these harms.

Many local areas have developed strategies to try and mitigate the adverse effects of Universal Credit,^27^ through providing advice and discretionary payments. Our evidence indicating the health consequences of Universal Credit for unemployed people highlights that it is essential that the health-care service engages as a key player in these local partnerships. Such engagement could involve ensuring that rapid access to mental health support is available, for example.

Universal Credit, although unique to the UK, represents a substantial change in the design and implementation of welfare benefits and has international relevance. Other countries considering such significant changes to their welfare system (eg, digitalised service, payment monthly in arrears, and stricter sanctions) should consider our results and other research that shows the negative mental health impact of systematic changes to the welfare system.^1^, ^22^ Furthermore, the mechanisms for the effect found in our study remain unclear. Further research should try to disentangle particular elements of this complex policy change to better design changes to welfare policy. This evidence is important internationally in the context of debates about the effectiveness of welfare assessment, conditionality, and sanctions.^6^

The adverse health effects of Universal Credit we report also potentially increase costs for the health-care service, social care, and welfare system. It is crucial that in assessing the costs and benefits of new welfare policies, policy makers take into account these consequences. Although the UK government has commissioned an evaluation of Universal Credit, the evaluation framework only includes an assessment of labour market outcomes; there is no plan to assess the effect on health and wellbeing.^28^ Given the evidence for the adverse health effects of welfare changes, as suggested by our analysis and previous studies, it is crucial that the UK government includes the robust evaluation of health impacts in its evaluation of Universal Credit and other welfare changes.

In summary, our analysis, alongside the growing body of evidence suggests that, in its current form, Universal Credit might have had a negative effect on the mental health of unemployed people, and actions to address this effect are needed to help tackle the UK mental health crisis.

## Data sharing

Our statistical code is available on request to the corresponding author.

## Supplementary Materials

Supplementary appendix

Supplementary Video Roll out of Universal Credit in the UK Unemployed people on Universal Credit as a percentage of all unemployment benefits, 2013–18. **Youtube url:**https://youtu.be/IUuU9xJtRaE
