# Supplementary material for: Effects on mental health of a UK welfare reform, Universal Credit: a longitudinal controlled study
Source: Lancet Public Health. 2020 Feb 27;5(3):e157–64. doi: 10.1016/S2468-2667(20)30026-8 (PMC7208537; doi:10.1016/S2468-2667(20)30026-8)
Supplement: Supplementary appendix [file mmc1.pdf]

THE LANCET
Public Health

Supplementary appendix

This appendix formed part of the original submission and has been peer reviewed. We post it as supplied by the authors.

Supplement to: Wickham S, Bentley L, Rose T, Whitehead M, Taylor-Robinson D, Barr B. Effects on mental health of a UK welfare reform, Universal Credit: a longitudinal controlled study. *Lancet Public Health* 2020; **5:** e157–64.

Table of Contents

Appendix 1. ***Different patterns of psychological distress as assessed by the General Health Questionnaire-12 (GHQ-12) over the different job definitions in Understanding Society dataset.*** **1**

Appendix 2. ***Flowchart of study population and sample size.*** **2**

Appendix 3. ***A brief description of Universal Credit and how it has been implemented over time.*** **3**

Figure E1. Timeline of main welfare changes in the UK between 2013 and 2018. .... *3*

Appendix 4. ***Description of the difference-in-difference method and equation for multivariable mixed effects linear regression model for psychological distress during a major policy change (introduction of Universal Credit).*** **4**

Figure E2. Graphical representation of parallel trends assumption in Difference-in-Difference analysis. *4*

Appendix 5. ***Full results of parallel trends analysis: Results from the multivariable logistic regression model for psychological distress (GHQ-12) based on analysis of the pre-policy period data to examine for the parallel trends assumption.*** **6**

Appendix 6. ***Full results of main analysis: Results from the multivariable logistic regression model for psychological distress (GHQ-12) based on analysis of 197,111 observations of 52,187 working age individuals*** **7**

Appendix 7. ***Robustness tests and additional analysis: Comparison of the parallel trends in psychological distress in the pre-policy period using alternative regression models are shown in the table below.*** **9**

References **11**

Appendix 1. *Different patterns of psychological distress as assessed by the General Health Questionnaire-12 (GHQ-12) over the different job definitions in Understanding Society dataset.*

As seen in this figure, individuals that identified as long-term sick had greater levels of adverse mental health effects prior to the onset of Universal Credit. It has been found that these groups of individuals were affected by other benefit changes to disability benefits which have been shown to have adverse mental health effects during this time and were therefore excluded from the analysis.(1)

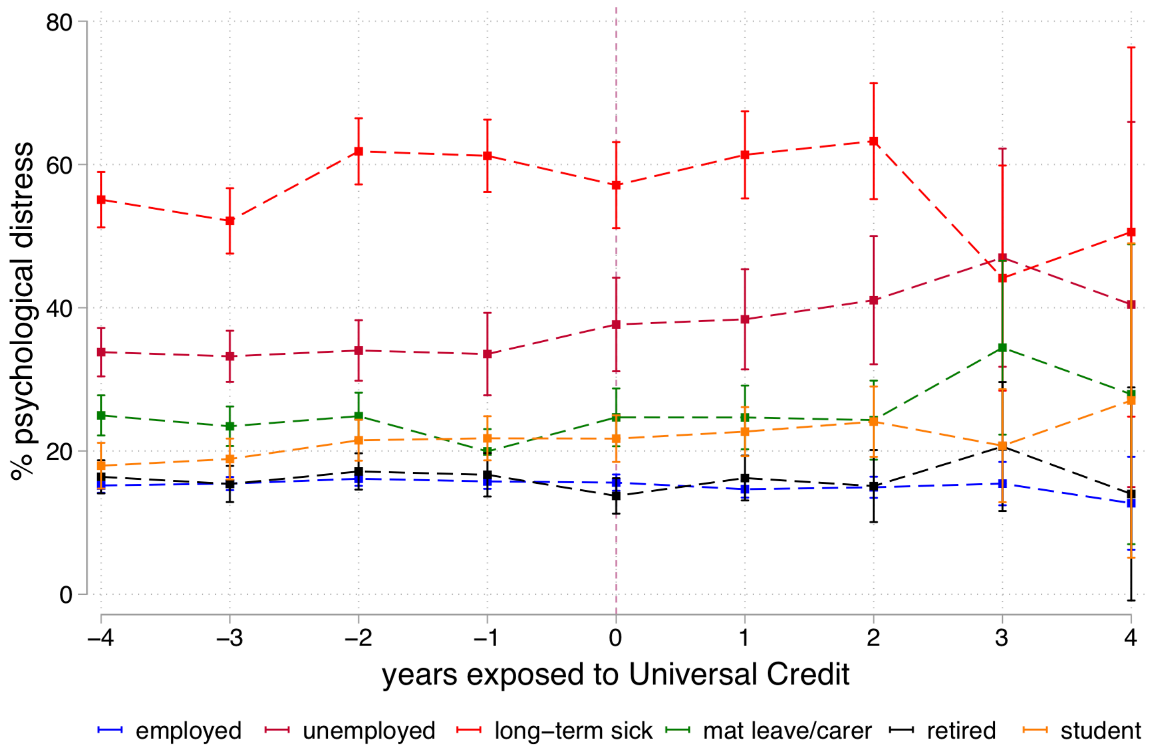

Appendix 2. *Flowchart of study population and sample size.*

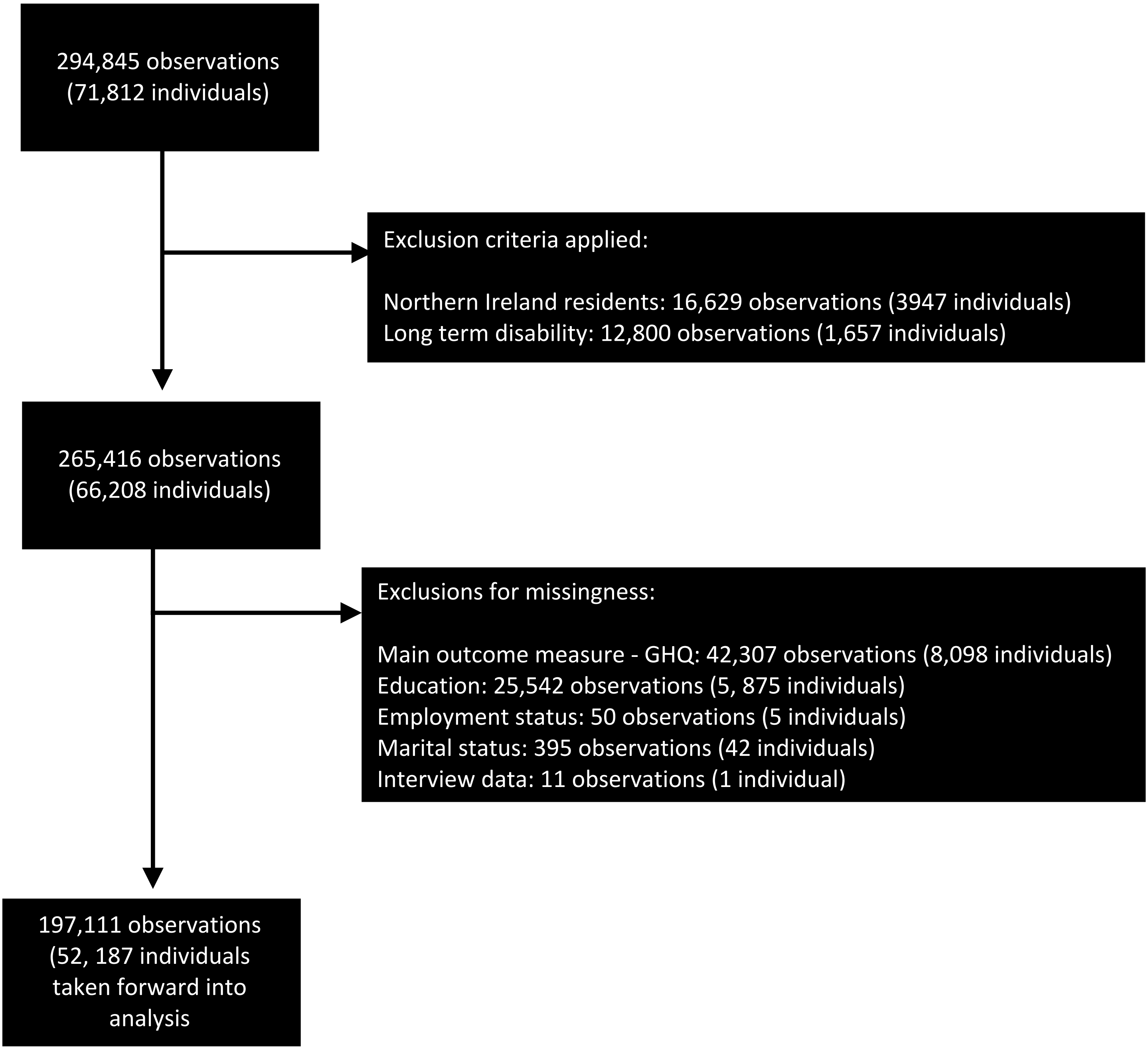

Appendix 3. *A brief description of Universal Credit and how it has been implemented over time.*

Universal credit is a means-tested benefit for people of working age who are on low or no income. It replaces six existing means-tested benefits. These include income support, income-based Jobseeker's Allowance, income -related employment and support allowance, housing benefit, child tax credit and working tax credit. (see figure 1 in main text)

Universal Credit pilots started in a 10 Jobcentres in the North West region of England in 2013 to 2014. In these pilot areas eligibility was restricted to new single then couple jobseekers, then some areas extended eligibility to claimants with children.

National roll-out started in 2015 to only new benefit claims by single jobseeekers, all other groups were expected to claim existing benefits. As of September 2018, new claims by all other groups were accepted onto Universal Credit. Individuals who do not have a change in circumstance and are on existing means-tested benefits, were not moved onto Universal Credit until July 2019. Currently those receiving severe disability premium are excluded from claiming Universal Credit.

In our analysis we have restricted the intervention group to unemployed individuals only, as over the study period they are the most likely to be affected based on the time scale of Universal Credit roll-out and transitions onto Universal Credit.

The figure below shows how Universal Credit was introduced in the context of other welfare changes over the same time period. As Universal Credit was introduced, it absorbed many of the other welfare changes that occurred at fixed time points (e.g. "bedroom tax", benefit cap, benefit freeze and two child policy).

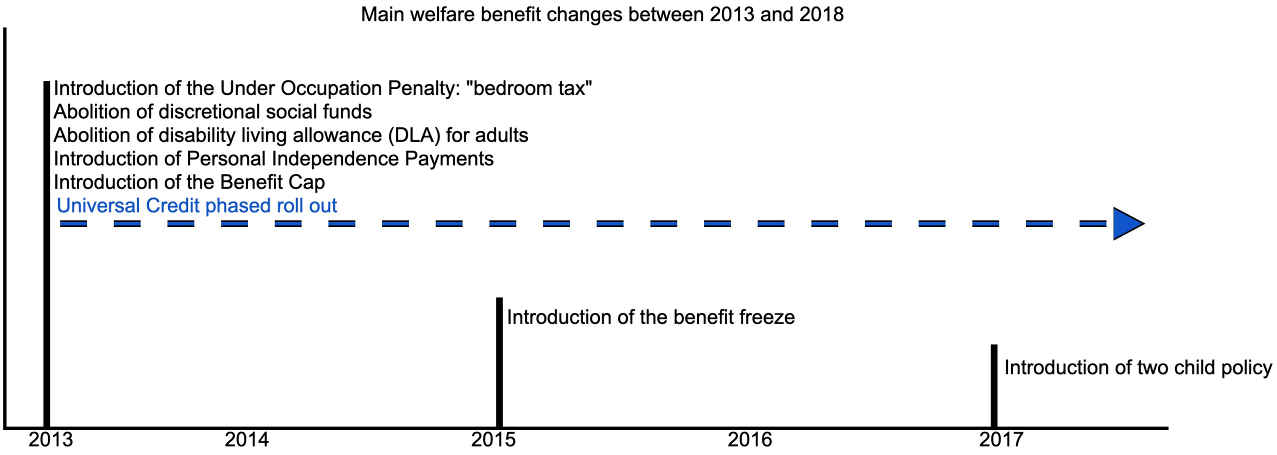

Figure E1. Timeline of main welfare changes in the UK between 2013 and 2018.

Appendix 4. *Description of the difference-in-difference method and equation for multivariable mixed effects linear regression model for psychological distress during a major policy change (introduction of Universal Credit).*

Difference-in-difference (DiD) analysis are an established approach used in econometrics (2,3) and are increasingly used in health research (4,5) for evaluating the impact of interventions, where the researcher has not manipulated the assignment of the intervention, sometimes known as "natural experiments"

A key assumption of the DiD method is the parallel trends assumption. That is, in the pre-intervention period the trends in the outcome remain parallel. If the assumption is true, the difference between the change in the outcome between the intervention and the comparison groups provides an unbiased estimate of the interventions effect.

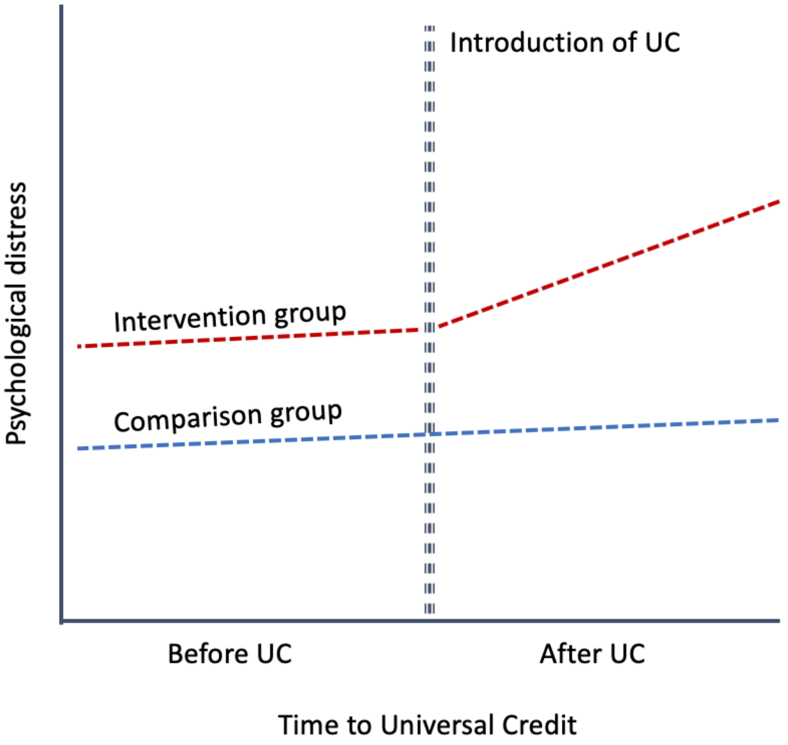

Figure E2. Graphical representation of parallel trends assumption in Difference-in-Difference analysis.

To test the parallel trends assumption has not been violated we can observe it graphically and with a regression model in the pre-intervention period only (Before UC was introduced). If the interaction term between the treatment group (intervention versus comparison) and Period (pre-policy period) are non-significant then we can assume they are parallel, and the assumption has not been violated. Therefore, DiD is appropriate.

The equation for the DiD in our main analysis is described below:

$$ln\left(\frac{P}{1-P}\right) = \beta_{0j} + \beta_1 Country_{ij} + \beta_2 Time_{ij} + \beta_3 Age_i + \beta_4 Age^2 ij + \beta_5 Sex_{ij} + \beta_6 Marital\ Status_{ij} + \beta_7 Education_{ij} + \beta_8 Treatment_{ij} + \beta_9 Period_{ij} + \beta_{10} Treatment_{ij} * Period_{ij} + \varepsilon_{ij}$$

Where $ln\left(\frac{P}{1-P}\right)$ is psychological distress (scored by GHQ-12) for individual *i* in year *j.* The intercept β_{0j} contains a random effect term and thus varies across years. *Country* represents the place people live in (England = 1, Scotland = 2, Wales = 3); Time represents the number of months exposed to Universal Credit; *Age* is the working age of participants (continuous measure from 16-64 years of age); we included age squared was also included in the model as this has a non-linear relationship with psychological distress; *Sex* is sex of participants (male = 1, female = 2); *Martial Status* is the marital status of participants (married = 0, unmarried = 1), *Education* is the highest educational attainment (degree or equivalent = 0, GCSE - A-levels or equivalent = 1, No or other qualification = 2); *Treatment* is a dummy variable indicating eligibility to receive the intervention (Comparison group = 0, Unemployed = 1); *Period* indicates whether year *j* occurs post or pre-intervention (post-intervention = 1; pre-intervention = 0); Treatment*Period is the DiD estimator; and ε *is the error term.*

Appendix 5. *Full results of parallel trends analysis: Results from the multivariable logistic regression model for psychological distress (GHQ-12) based on analysis of the pre-policy period data to examine for the parallel trends assumption.*

Mental health effect	OR	Std Error	t	P value	Lower 95%CI	Upper 95% CI
Age	0.006	0.001	8.030	<0.0001	0.005	0.008
Age squared	-0.000	0.000	-7.910	<0.0001	-0.000	-0.000
Country						
Scotland	-0.011	0.007	-1.570	0.117	-0.025	0.003
Wales	-0.010	0.007	-1.380	0.166	-0.025	0.004
Unmarried	-0.050	0.004	-11.950	<0.0001	-0.059	-0.042
Sex						
female	0.066	0.003	20.070	<0.0001	0.059	0.072
Education						
GCSE-A levels	0.001	0.004	0.200	0.842	-0.007	0.009
Other/No qual	0.005	0.005	0.990	0.320	-0.005	0.015
Unemployment	0.182	0.024	7.710	<0.0001	0.136	0.228
Year to UC	0.003	0.001	2.500	0.012	0.001	0.005
Treatment#Time						
Unemployed#months before policy introduction	0.008	0.006	1.380	0.168	-0.003	0.019
_cons	0.052	0.015	3.550	<0.0001	0.024	0.081

Appendix 6. *Full results of main analysis: Results from the multivariable logistic regression model for psychological distress (GHQ-12) based on analysis of 197,111 observations of 52,187 working age individuals*

Mental health diff	OR	Linearized Std Error	t	P value	Lower 95%CI	Upper 95% CI
Year of UC	1.018	0.007	2.610	0.009	1.005	1.032
Age	1.038	0.005	7.900	<0.0001	1.028	1.048
Age squared	1.000	0.000	-7.670	<0.0001	0.999	1.000
Country						
Scotland	0.905	0.042	-2.170	0.030	0.827	0.990
Wales	0.982	0.047	-0.390	0.698	0.894	1.078
Unmarried	0.677	0.016	-16.230	<0.0001	0.646	0.710
Sex						
Female	1.619	0.035	22.390	<0.0001	1.552	1.689
Education						
GCSE-Alevels	1.032	0.027	1.230	0.218	0.981	1.086
Other/No qual	1.052	0.035	1.540	0.124	0.986	1.123
Unemployed	2.381	0.100	20.650	<0.0001	2.193	2.585
Post Intervention	0.934	0.038	-1.660	0.097	0.862	1.012
Treatment#Period						
Unemployed#Post	1.382	0.153	2.920	0.003	1.112	1.718
_cons	0.095	0.009	-25.280	<0.0001	0.079	0.114

Marginal test

	DiD estimator	Std Err	t	P value	Lower 95%CI	Upper 95% CI
Treatment#Period (1 vs 0) (1 vs 0)	0.066	0.025	2.640	0.008	0.017	0.114

The number of new claims for UC amongst unemployed people between April 2013 and December 2018 was 1,020,351 (source DWP statistics).(6) Based on our estimate that going onto UC increased the prevalence of psychological distress of 6.6 percentage points (based on marginal analysis above, we estimate that and additional 6.6% of these people developed psychological distress than would have been the case in the absence of UC. This provides the estimate of an additional 6.6% x 1,020,351= 63,674. Therefore roughly 64,000 people have experienced psychological distress as a result of the policy introduction [95% CI 10,042 to 117,307].

Note the number of new claims for UC amongst unemployed people between April 2013 and December 2018 data takes the number of people on Universal Credit relative to the second Thursday of each month with the count of the number of UC claims at the end of each quarter that have a duration of under three months to approximate the number of new starts to UC. It

is possible that these data may both underestimate and overestimate the true count as they as won't count people who started then stopped before the end of the quarter, but they may also overestimate due to the possibility that some people may make repeat claims.

Appendix 7. *Robustness tests and additional analysis: Comparison of the parallel trends in psychological distress in the pre-policy period using alternative regression models are shown in the table below.*

Note for model 5: Imputations of 10 datasets were performed using the analytical models in Stata 14 (command: mi estimate). All variables included in the main analysis were included in the imputation process. The multiple imputation of the 10 datasets adjusts coefficients and standard errors for the variability between imputations according to the combination rules set by Rubin (1987). (7)

Alternative specifications models[1]		Coefficient (95% CIs)	Lower 95% CI	Upper 95% CI	p-value
1	Linear probability regression model	0.008	-0.003	0.019	0.163
2	Removing employed people from comparison sample	0.005	-0.007	0.017	0.385
3	Restricting analysis to those with only new onset of unemployment and psychological distress	-0.005	-0.039	0.029	0.767
4	Using a balanced panel	0.005	-0.014	0.025	0.591
5	Using multiple Imputations*	0.026	-0.028	0.080	0.346

Comparing difference-in-difference estimator using alternative regression models are shown in the table below.

Alternative specifications models[1]		OR (95% CIs)	Percentage point change in prevalence of psychological distress	Lower 95% CI	Upper 95% CI	p-value
1	Linear probability regression model	-	7.71%	3.75	12.66	0.002
2	Removing employed people from comparison sample	1.25 (0.10, 1.58)	4.32%	-0.57	9.21	0.080
3	Restricting analysis to those with only new onset of unemployment and psychological distress	1.73 (1.01,2.95)	12.15%	-0.39	24.68	0.058
4	Using a balanced panel	1.53 (1.17,2.01)	10.25%	3.00%	15.49%	0.004
5	Using Multiple Imputations	1.38 (1.22,1.73)	6.5%	1.61	11.33	0.009

[1]Each model controlled for age, age squared, sex, education, country, and marital status.

To investigate whether there were differential effects across subgroups within the main model we tested the interaction of age with the intervention by period interaction term. We repeated this for sex and education in three separate logistic regression models. Results are in the table below

	Interaction measures		OR	Lower 95% CI	Upper 95% CI	P value
1	Age# treatment # policy interaction	16-24	-	-	-	-
		25-44	0.97	0.55	1.73	0.927
		45-54	0.82	0.47	1.43	0.478
		55-64	0.55	0.26	1.16	0.116
2	Sex # treatment # policy interaction	Male	-	-	-	-
		Female	1.11	0.73	1.70	0.630
3	Education # treatment # policy interaction	Degree or higher	-	-	-	-
		GCSE/A-levels or equiv	1.29	0.75	2.24	0.359
		Below GCSE/other	1.03	0.58	1.85	0.912

Additional analysis not described in main text

Replacing Country covariate with Region:

highq	Odds Ratio	Std Error	t	P- value	Lower 95%CI	Upper 95% CI
Year of UC	1.021	0.007	3.010	0.003	1.007	1.035
Age	1.037	0.005	7.700	<0.0001	1.027	1.046
Age squared	1.000	0.000	-7.480	0.000	0.999	1.000
GOR						
Wales	0.903	0.145	-0.640	0.525	0.659	1.237
Scotland	0.980	0.158	-0.120	0.902	0.715	1.344
East Midlands	0.979	0.157	-0.130	0.894	0.714	1.342
East of England	0.915	0.143	-0.570	0.570	0.673	1.244
London	0.928	0.147	-0.470	0.638	0.681	1.266
North East	1.000	0.157	0.000	0.998	0.735	1.362
North West	1.025	0.162	0.160	0.875	0.753	1.396
South East	1.004	0.158	0.030	0.978	0.738	1.367
South West	1.102	0.176	0.610	0.541	0.806	1.507
West Midlands	1.024	0.160	0.150	0.878	0.754	1.392
Yorkshire and the Humber	0.954	0.150	-0.300	0.764	0.701	1.298
Unmarried	0.678	0.016	-16.320	<0.0001	0.647	0.711
Sex						
Female	1.613	0.035	22.280	<0.0001	1.547	1.683
Education						
GCSE-A levels	1.045	0.027	1.720	0.085	0.994	1.098
Other/No qual	1.066	0.035	1.930	0.054	0.999	1.137
Unemployed	2.383	0.100	20.620	<0.0001	2.194	2.588
Post Intervention	0.931	0.038	-1.750	0.080	0.859	1.009
Treatment#Period						
Unemployed#Post	1.383	0.153	2.930	0.003	1.113	1.717
_cons	0.097	0.017	-13.080	<0.0001	0.069	0.138

References

1. Barr B, Taylor-Robinson D, Stuckler D, Loopstra R, Reeves A, Whitehead M. 'First, do no harm': are disability assessments associated with adverse trends in mental health? A longitudinal ecological study. Journal of Epidemiology and Community Health. 2015;70(4):jech-2015-206209.

2. Angrist JD, Pischke J-S. Mostly Harmless Econometrics: An Empiricist's Companion. Princeton: Princeton University Press 2009.

3. Wooldridge JM. Econometric Analysis of Cross Section and Panel Data. 2nd Revised ed. Cambridge: Mass: MIT Press 2010.

4. Craig P, Dieppe P, Macintyre S, et al. Developing and evaluating complex interventions: the new Medical Research Council guidance. BMJ 2008;337:a1655. doi: 10.1136/bmj.a1655 [published Online First: 2008/10/01]

5. Craig P, Katikireddi SV, Leyland A, et al. Natural Experiments: An Overview of Methods, Approaches, and Contributions to Public Health Intervention Research. Annu Rev Public Health 2017;38:39-56. doi: 10.1146/annurev-publhealth-031816-044327 [published Online First: 2017/01/27]

6. Department for Work and Pensions. Stat-Xplore. https://stat-xplore.dwp.gov.uk/ Department for Work and Pensions. 2019.

7. Rubin, D. B. 1987. Multiple Imputation for Nonresponse in Surveys. New York: Wiley.
